# Laboratory and field evaluation of MAA, an ointment containing *N*,*N*-diethyl-3-methylbenzamide (DEET) against mosquitoes in Burkina Faso

**DOI:** 10.1186/s12936-021-03755-6

**Published:** 2021-05-20

**Authors:** Alphonse Traor, Grard Niyondiko, Antoine Sanou, Franck Langevin, NFal Sagnon, Adama Gansan, Moussa Wamdaogo Guelbeogo

**Affiliations:** 1 Centre National de Recherche et de Formation sur le Paludisme, 01 BP 2208 Ouagadougou 01, Burkina Faso; 2 100000 vies, 12 BP 499 Ouagadougou 12, Burkina Faso

**Keywords:** Malaria, Mosquito, *Anopheles gambiae*, *Aedes aegypti*, Repellent, MAA, Burkina Faso

## Abstract

**Background:**

Malaria vector control relies upon the use of insecticide-treated nets and indoor residual spraying. However, as the emergency of insecticide resistance in malaria vectors grows, the effectiveness of these measures could be limited. Alternative tools are needed. In this context, repellents can play an important role against exophagic and exophilic mosquitoes. This study evaluated the efficacy of MAA, a novel repellent ointment, in laboratory and field conditions in Burkina Faso.

**Methods:**

For laboratory and field assessment, 20 volunteers were enrolled and trained for nocturnal collection of mosquitoes using human landing catches (HLC). In the laboratory tests, 2mg/sq cm of treatment (either MAIA or 20% DEET) were used to assess median complete protection time (CPT) against two species: *Anopheles gambiae* and *Aedes aegypti*, following WHO guidelines. For both species, two strains consisting of susceptible and local strains were used. The susceptible strains were Kisumu and Bora Bora for *An. gambiae* and *Ae. aegypti*, respectively. For the field test, the median CPT of MAA was compared to that of a negative (70% ethanol) and positive (20% DEET) after carrying out HLCs in rural Burkina Faso in both indoor and outdoor settings.

**Results:**

Laboratory tests showed median Kaplan-Meier CPT of 6h 30min for *An. gambiae* (Kisumu), 5h 30min for *An. gambiae* (Goden, local strain), and 4h for *Ae. aegypti* for both the local and sensitive strain. These laboratory results suggest that MAA is a good repellent against the three mosquito species. During these field tests, a total of 3979 mosquitoes were caught. In this population, anophelines represented 98.5%, with culicines (*Aedes*) making up the remaining 1.5%. Among anopheline mosquitoes, 95% belonged to the *An. gambiae* complex, followed by *Anopheles funestus* and *Anopheles pharoensis*. The median CPT of 20% DEET and MAA were similar (8h) and much longer than that of the negative control (2h).

**Conclusions:**

Results from the present studies showed that MAA offers high protection against anophelines biting indoors and outdoors and could play an important role in malaria prevention in Africa.

## Background

Malaria is one of the deadliest diseases in many low- and middle-income countries, affecting mainly children and pregnant women in sub-Saharan Africa [1]. Long-lasting insecticide-treated nets (LLINs) have been regarded as the most effective method for controlling mosquitoes transmitting malaria parasites. Since 2000, about one billion nets have been distributed in Africa, resulting in a significant decline in malaria-related deaths on the continent between 2000 and 2015 [2,4].

However, the massive use of insecticides in public health in addition to that in agriculture causes concern regarding insecticide resistance [5,7] and changing behaviour [8, 9] of the malaria vectors. For example, a study conducted in Papua New Guinea showed a shift in mosquito biting from night to earlier hours in the evening after a nationwide distribution of LLINs [10]. Similar changes in the behaviour of *Anopheles funestus* have been observed in Benin and Senegal after LLIN distribution achieved a high level of coverage [9, 10]. Furthermore, studies suggest that the scaling up of LLIN distribution and indoor residual spraying (IRS) have led to more outdoor biting by *Anopheles gambiae sensu lato* (*s.l.*), commonly considered endophagic mosquitoes [11,13]. A recent study in the Cascades region of Burkina Faso showed a high level of insecticide resistance [14] where more than 50% of the major vector, *An. gambiae s.l.*, were collected biting outdoors [15]. These altered patterns of outdoors, early evening and morning biting, by anophelines, combined with resistance to insecticides appear to be caused by the mass distribution of LLINs and imply the inexorable loss of efficacy of these interventions [16, 17]. A recent study highlighted that an increase in early evening biting could increase transmission not only because people are unprotected by nets, but also because there is a higher chance of malaria vectors becoming infectious [18]. The development of new vector control tools, in addition to LLINs, is therefore necessary to protect people, when they are not under a bed net.

Topical repellents could play an important role in addressing this problem if they are effective and accepted by the population. A systematic review of repellent interventions and mathematical modeling has shown that user compliance is indeed one of the most decisive factors for the success of this intervention [19]. In sub-Saharan Africa, ointments are used primarily by mothers and children to moisturize their skins. In Burkina Faso, ointments are applied to 80% of children every evening, when mosquitoes start biting (Kadidia Ouedraogo et al., in prep). Maa Africa, a company based in Burkina Faso, has developed a mosquito repellent ointment, MAA, uniquely designed with local mothers, to be used daily within their families. The underlying idea is to leverage the existing habits of mothers to protect their families from infectious bites whenever they are not under a net. Affordability is a key criterion for the products adoption and use. MAA has been industrially produced since June 2020 in Cte dIvoire and integrates a large share of ingredients sourced in West Africa. The ointment was officially launched in August 2020 in Burkina Faso and over 50,000 units were sold in the first four months. In March 2021, over 500 points of sales (mainly general stores, pharmacies and kiosks) distributed the product in the country. If MAA proves that it is both effective and accepted by the population, it could play a key role in reducing the probability of children experiencing infectious bites during the evening and be positioned as a complementary intervention to LLINs.

The aim of this study is to evaluate the effectiveness of MAA in both laboratory and field conditions, especially the median complete protection time (CPT) offered by the product. Results from these evaluations are important for validating how effective this new repellent is; behavioral responses to repellent differ between wild mosquito and laboratory-reared mosquito populations [20].

## Methods

### Study area

**Fig. 1:**
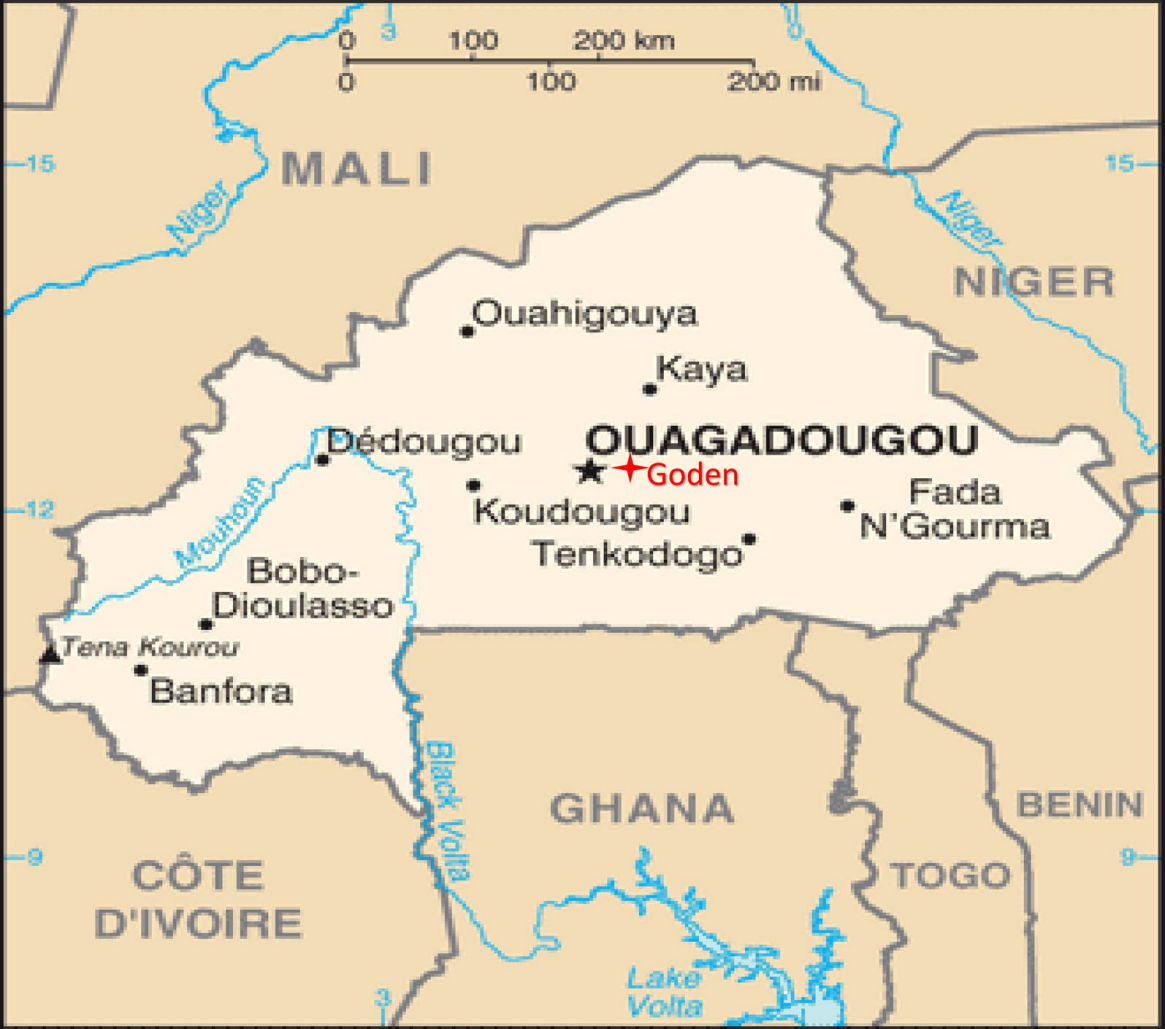
Study area

Laboratory tests were conducted in May 2019 in the insectary of *Centre National de Recherche et de Formation sur le Paludisme* (CNRFP) in Ouagadougou, Burkina Faso. Field tests were carried out at Goden (1225N, 120W), a site located at 15km northeast of Ouagadougou, the capital city of Burkina Faso (Fig.1). Goden is a rural village with a Sudanian savanna climate and rainfall under 900 mm annually. The ~800 inhabitants mainly belong to the Mossi ethnic group, and are mostly devoted to agriculture and raising pigs, dogs, goats, and chickens within their compounds. LLINs were distributed in 2016 to ~90% of the population. Goden is known for its high density of malaria vectors due to its proximity to the Massili River. The field study was carried out during the rainy season (August to November 2019) corresponding to high vector density and high malaria transmission. A preliminary assessment of the mosquito density on the collection site was carried out using human landing catches (HLCs) before the tests started.

### Human volunteer preparation

Healthy adult male volunteers aged between 18 and 40 years were enrolled in this study. The volunteers were instructed not to use fragranced soaps, perfume, tobacco, or alcohol 12h before the start and throughout their participation. To establish the amount of repellent required for application, the surface area of the arm (for laboratory tests) or the leg (for field tests) of volunteers was determined using the following formula: Area= (C_w_+ C_e_) D_we_.

Here C_w_ is the circumference of the wrist or ankle in cm, C_e_ is the elbow cubital fossa or the knee circumference in cm, and D_we_ is the distance in cm between C_e_ and C_w_ [21]. The amount of ointment needed for each volunteer was determined depending on the area of their forearm or leg length. The quantity of product left in bottles was weighed using a precision weighing balance (KERN & SOHN GmbH, Balingen, Germany) to determine the amount applied by each volunteer.

### Repellents

MAA, a shea butter-based ointment containing 15% DEET (*N*, *N*-diethyl-3-methylbenzamide) was received from Maa Africa SAS. It was tested against an ethanolic solution of 20% DEET as positive control and a negative control of 70% ethanol. DEET is known as the standard repellent reference.

### Strains of mosquitoes

Four strains of mosquitoes were used in the laboratory tests, including Kisumu F57 and Bora bora F58 susceptible strains of respectively *An. gambiae* and *Aedes aegypti*. In addition, local strains laboratory-colonized from rural areas of Goden, in Burkina Faso, were used: hereafter named *An*-Goden (*An. gambiae* local strain, F418) and Loc-*Aedes* (local *Ae. aegypti*, F318). These species were maintained under a 12:12h (light: dark) photoperiod. During rearing, larvae were fed on fish food while glucose was used for adults. The temperature and relative humidity in the rearing room were 2528C and 6080%, respectively. Individual mosquitoes used in these experiments were 5 to 10 days old nulliparous females starved from sugar solution for 12h before the experiment.

### Evaluation in the laboratory

The laboratory experiments were conducted following WHO guidelines for the arm-in-cage test [21]. Cages were 454545cm screen enclosures. Two test cages were used, one for the repellent candidate and the other for the positive control. The test cages contained 200 females aged 5 to 10 days of one of the four mosquito strains: Kisumu, *An-*Goden, Bora bora and loc-*Aedes*. The experiment in the laboratory was carried out at temperatures ranging between 25 and 28C, with relative humidity between 60 and 80%.

Overall, 2mg of ointment were applied per sq cm on the left forearm of each volunteer, a concentration estimated from the average of 5 volunteers from the laboratory asked to apply ointment on their left forearm as they would normally do in real life. In all subsequent repellent trials, volunteers were then supplied with a total volume that would achieve this concentration over the surface area of their forearms and/or legs (determined as described above). A steel spatula was used to apply the ointment on the forearm of each volunteer prior to each experiment to avoid absorbing ointment on non-targeted areas of skin. Positive control consisted of 1 ml of 20% DEET solution applied to the right forearms of volunteers.

Negative controls and MAA test arms were prepared by first washing left forearms with odorless soap, drying and rinsing with 70% ethanol solution and then drying again. All volunteers wore latex gloves to protect their hands from mosquitoes. To assess the readiness of the mosquitoes to land, both left and right cleaned forearms of volunteers were exposed in the experimental cages for 30s (or until 10 landings of mosquito were counted). Then, for each volunteer, the right forearm was treated from wrist to elbow using 1 ml of the 20% DEET solution whilst the left forearm was treated from the wrist to elbow with MAA ointment. Thirty mins after application of the repellents, the volunteer exposed their treated forearm in the test cage for 3min. The procedure was repeated every 30min until the first bite occurred and the elapsed time to the first bite was recorded. The test was performed three times for each volunteer per mosquito species. Considering the difference in the relative periods of biting activity of each mosquito species, the tests using *Ae. aegypti* strains were carried out between 09:00 and 18:00, whereas those for *An. gambiae* were conducted between 17:00 and 05:00 [21].

### Field evaluation

The lower legs of volunteers were washed with neutral soap, rinsed with 70% of ethanol solution and naturally dried. Once their legs were treated volunteers were asked to avoid rubbing, touching or wetting the repellent-treated area. Two mg of MAA per sq cm (2.40.2g per 118979.2 sq cm) and 2 ml per sq cm of 20% DEET (2 ml0.1 ml per 118979.2 sq cm, as a positive control) were applied to volunteers lower legs, from knee to ankle. A total of 20 volunteers were recruited from Goden village and trained for nocturnal mosquito collection using HLC. Each volunteer was later randomly allocated to one of the five groups (2 for MAIA, 2 for positive control, 1 for negative control) of four volunteers according to the repellent received. Each night of collection, the experiment took place at five different households, located at least 20m apart as per WHO guidelines [21], in order to avoid biases in attractiveness to the mosquitoes.

Mosquito collection started 30min following treatments. Volunteers acting as bait, sat on a chair in pairs (one indoor and one outdoor) and actively collected mosquitoes that landed on their treated lower leg using mouth aspirator and flash torch [22] for 45min, followed by a 15-mins break. Volunteers wore long-sleeved shirts, buttoned at the wrist, long trousers, closed shoes and latex gloves with a hat on their head, but with the treated lower leg to be exposed to mosquitoes by rolling up trousers to the knee. During these experiments, mosquitoes were collected simultaneously indoors and outdoors between 19:00 and 06:00. To avoid biases introduced by individual attractiveness and skills [23, 24] volunteers at the same household rotated between indoors and outdoors hourly. In each household two groups of two people rotated collecting from 18:00 to 24:00 and from 00:00 to 06:00, following the Williams balanced Latin Square design.

Collected mosquitoes were transferred into plastic cups, covered with a piece of untreated net, with a small hole at the bottom to allow mosquitoes to be easily aspirated into them. After collection mosquitoes were brought to the entomological laboratory of CNRFP and morphologically identified using a stereo microscope and identification keys [25].

### Side effects

No side effects were observed or reported by any of the volunteers throughout the period of tests both in the laboratory and in the field.

### Ethical clearance

Written informed consent was obtained from all volunteers and household owners recruited in this study. The study was approved by the institutional ethic committee of CNRFP under 2019/000008/MS/SG/CNRFP/CIB.

### Data analysis

All data were collected on standard forms and entered twice in a database by different people. Databases have been compared using Epi Info 3.5.3, and inconsistencies were verified using printed and corrected forms. The performance of the repellent was measured by calculating the repulsive efficiency and the median full protection time. A generalized linear mixed model (GLMM) was used to further analyse the effect of the location (indoors vs. outdoors) on the performance of the treatments. Variation in the average number of bites received between treatments was also assessed.

The median CPT is defined as the interval of time between the beginning of collection/test and the first mosquito landing. To estimate the median CPT of each treatment, a Kaplan-Meier survival analysis was performed for each vector species and strain used in the laboratory experiments and on field data through survival function from R software-version 3.5.0 (2018-04-23). However, for the field test, the analysis was performed on only *An. gambiae s.l.* as it was the most abundant species collected (~96% of the total collection). The analysis consisted of assessing the median CPT and the repulsive efficacy. The repulsive efficacy was calculated as a percentage of repulsion (% R) according to the formula % R = ((C T) / C) 100, where C is the number of mosquitoes collected on the treated legs of the two control treatments separately, and T is the total number of mosquito bite attempts on the volunteers legs treated with the test product [21].

## Results

### Laboratory tests

**Fig. 2:**
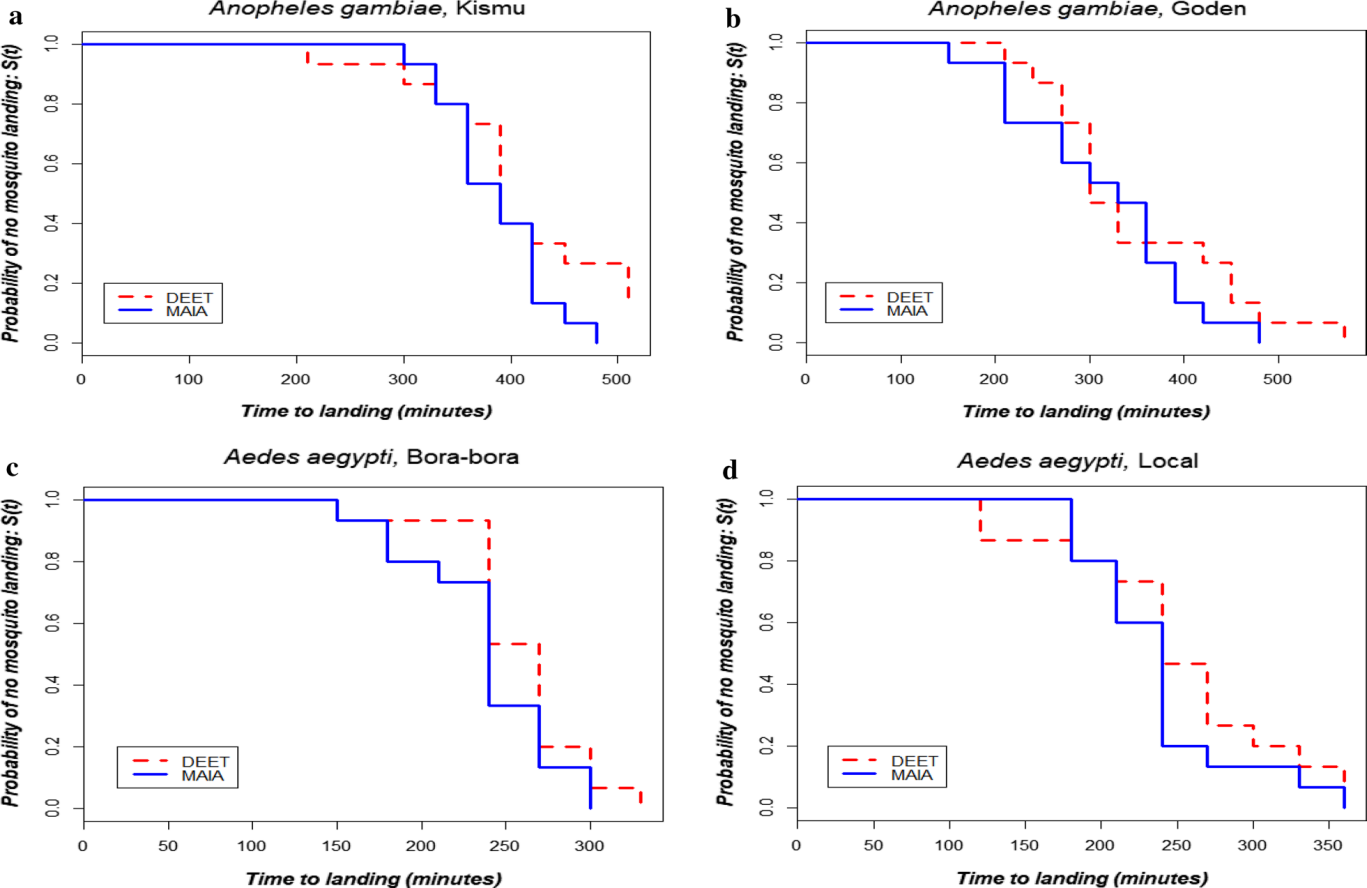
Kaplan-Meier plots for 20% DEET and MAA tested against the four species on five volunteers

Overall, under laboratory conditions the relative repellency (median CPT) was higher for both MAA and 20% DEET against *An. gambiae* compare to *Ae. aegypti* (Table1). MAA performed well in repelling the four mosquito species used in this study. The median CPTs were, respectively, 6.5h for *An. gambiae* (Kisumu), 5.5h for *An. gambiae* (Goden, local strain) and 4h for *Ae. aegypti* for both the local and sensitive strain. There was no significant difference between the two treatments for each of the experiment (Kisumu: ^2^=2.1, p value=0.14; Goden: ^2^=0.8, p value=0.36; Bora bora: ^2^=1.7, p value=0.19; *Ae. aegypti* (local strain): ^2^=0.9, p value=0. 35) indicating that both MAA and 20% DEET have equal repellency for these strains. The Kaplan-Meier curves for MAA and 20% DEET, respectively, for Kisumu, *An-*Goden, Bora bora and Loc-*Aedes* are shown in Fig.2.

**Table 1:** Median complete protection times (CPT) in minutes and their 95% confidence intervals (CI) against mosquito strains, according to treatments 20% DEET and MAA, under laboratory conditions

	*An. gambiae* Kisumu		*An. gambiae* Goden		*Ae. aegypti* Bora bora		*Ae. aegypti* Local	
	DEET	MAA	DEET	MAA	DEET	MAA	DEET	MAA
Median. CPT	390	390	300	330	270	240	240	240
Lower CI	368	334	272	216	252	239	212	225
Upper CI	412	446	328	444	288	241	268	255

### Field test

#### Mosquito species composition and biting behaviours

A total of 3,979 mosquitoes, stratified by treatment and species (Table2), were caught using HLC. Anophelines represented 98.5% of the total catch, with culicines (*Aedes*) making up the remaining 1.5%. Among anopheline species, 99.6% belonged to *An. gambiae* complex, followed by *An. funestus* (0.1%), and *Anopheles pharoensis* (0.3%). The frequency of mosquitoes landing on treated collectors, compared with control subjects, varied according to the repellent used (Table2). The hourly mosquito biting rate varied significantly between treatments (df=2, ^2^=426.22, p<0.0001). An average of 0.68 (95% CI: 0.510.91) mosquito bites were received per person per hour for MAA compared to 1.01 (95% CI: 0.761.33) for 20% DEET, and 8.98 (95% CI: 6.5612.29) for the 70% ethanol. In addition, there was no variation between treatments according to location (outdoors and indoors, df=2, ^2^=1.703, p=0.42). Overall, the ratio outdoors:indoors biting was 1.26 (95% CI: 1.251.27) showing that more bites were taking place outdoors compared to indoors (df=1, ^2^=5.79, p=0.016).

**Table 2:** Total number of common mosquitoes collected after treatment of 20% DEET, MAA and ethanol 70%

Mosquito species	Treatment		
	DEET	Ethanol	MAA
*Anopheles gambiae sensu lato (s.l.)*	686	2660	480
*Anopheles funestus*	1	2	1
Other *Anopheles*	1	9	3
Culicines	7	106	23

#### Repellency against mosquitoes

**Fig. 3:**
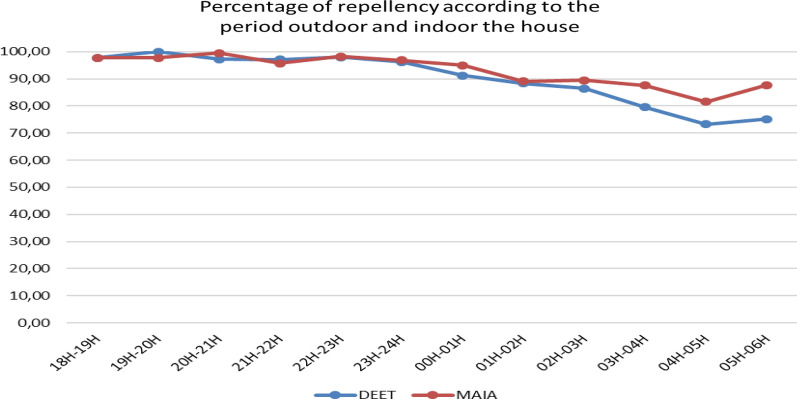
Repellency of 20% DEET and MAA indoor and outdoor collection

Repellency against *An. gambiae s.l.* was stratified by time of collection. From 18:00 to 24:00 (6h after application), the percentage of repellency varied from 100 to 90% for MAA and DEET. Between 00:00 to 03:00 (9h after application), the percentage was between 90 and 80% (Fig.3). After 03:00 (10h after application), this percentage was under 80% for 20% DEET, but MAA was over 80%. MAAgave a high percentage of repellency thoughout, however during the first 9h after applications no difference in the repellency was observed between MAA and the positive control.

When data were stratified by location of mosquito biting, the trend was the same for indoors and outdoors. No difference was observed during the first 9h between MAA and 20% DEET. These results show that MAA can protect both indoors and outdoors.

#### Complete protection time

**Fig. 4:**
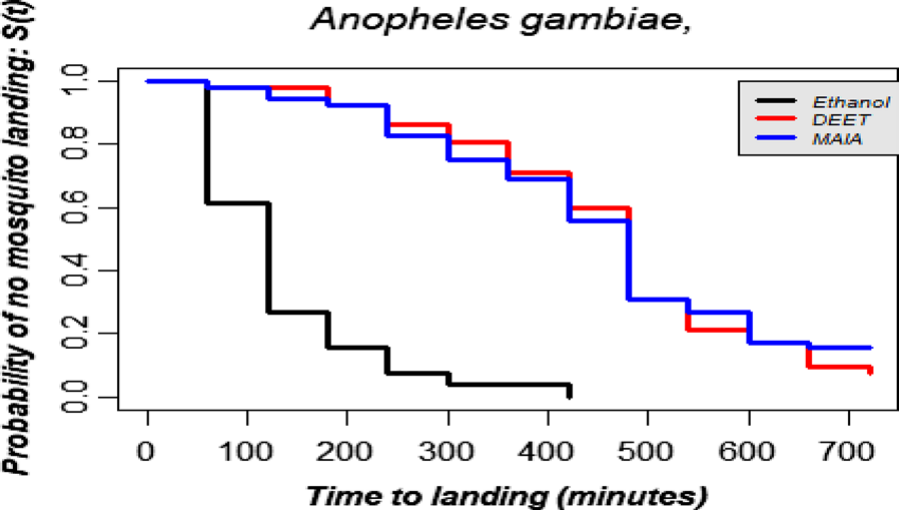
Overall estimated probabilities of no mosquitoes landing for each treatment according to time of collections

**Fig. 5:**
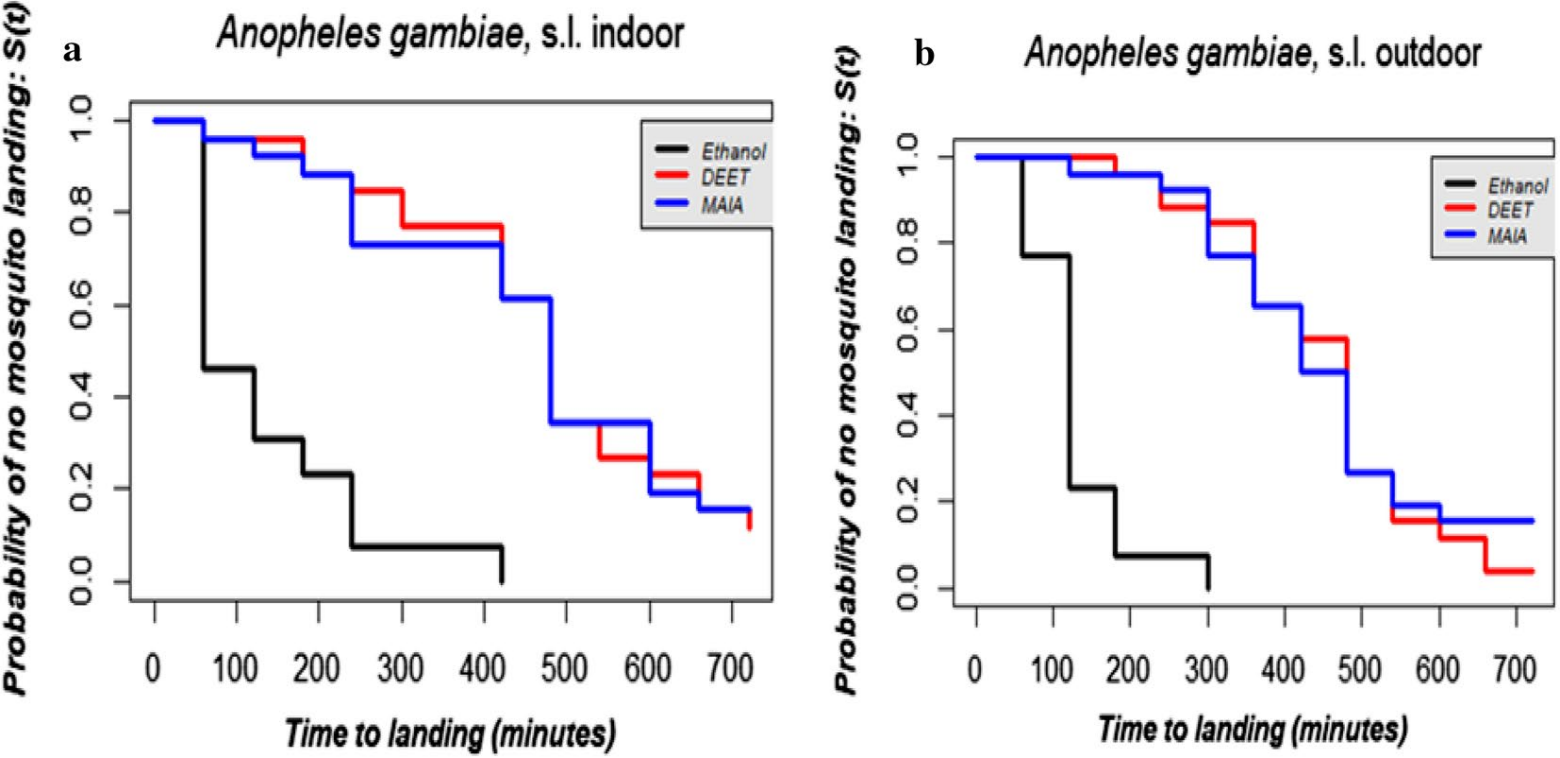
Estimated probabilities of no mosquitoes landing for each treatment according to the time at indoor (**A**) and outdoor locations (**B**)

The overall median CPTs of 20% DEET and MAA (Table3) were estimated at 480min (8h) against 120min (2h) for the negative control. For outdoor collections, the median CPTs were 480, 450 and 120min for 20% DEET, MAA and negative control respectively (Table4). For indoor collections, these estimates were 480, 480 and 60min, respectively, for 20% DEET, MAA and ethanol (Table4). Statistical analyses showed that there was no difference in the median CPT between 20% DEET and MAA (df=1, ^2^=0.2, p=0.7). However, there was a significant difference between median CPT as estimated for MAA and the negative control (df=2, ^2^=106, p<0.0001, Fig.4). Even when the collection was stratified by location this difference still occurred in both indoor (Fig.5A; df=2, ^2^=41.6, p<0.0001) and outdoor collections; df=2, ^2^=66.7, p<0.0001) Fig.5B).

**Table 3:** Estimated complete protection time (mins) with 95% CI, against *Anopheles gambiae s.l.* for 20% DEET, MAAand ethanol 70%

	*Anopheles gambiae s.l.*		
	DEET	Ethanol	MAA
Median CPT	480	120	480
Lower CI	454	91	448
Upper CI	506	149	512

**Table 4:** The estimated complete protection times (mins) with 95% CI, against *Anopheles gambiae s.l.* for 20% DEET, MAA and ethanol 70%, indoors and outdoors

	*Anopheles gambiae s.l*					
	Indoors			Outdoors		
	20% DEET	Ethanol	MAA	20% DEET	Ethanol	MAA
Median CPT	480	60	480	480	120	450
Lower CI	440	<60	440	447	95	428
Upper CI	521	NA	521	513	145	472

## Discussion

The results of this study demonstrated that MAA, a shea butter-based ointment with 15% DEET provides high protection against mosquitoes in Goden, a rural area of Burkina Faso. Both tests under field and laboratory conditions suggested that MAA has equal repellency effect as 20% DEET ethanolic solution during the period of collection. Similar results were also found both indoors and outdoors. The percentage of repellency when using MAA varied between 100 and 80% over the major malaria vector biting period, which occurs between 18:00 and 06:00. The median CPT were also similar and estimated around 480min. Both MAA and the 20% DEET ethanolic solution were found to provide up to 90% of repellency during the first 6h after their applications. Furthermore, results suggested that using MAA, the average hourly bites received was significantly lower (less than 1 bite per hour) compared to that of both 20% DEET and negative control. However, one of the limitations is that biting activity on control arms was not checked at the end of laboratory experiments to validate consistency of mosquito avidity, but this did not affect the overall result. Overall, it can be argued that MAA could provide protection to people before they go to bed.

Previous studies in the same locality comparing repellency between three different repellents found that DEET, IR3535 and KBR 3023 were effective against *An. gambiae s.l*. and other Afrotropical vector mosquitoes [26]. In this study authors showed that protection from KBR 3023, DEET and IR3535 were still high against anophelines for up to 10h post-exposure. In contrast, results from the current study indicated that the relative repellency was 100% for ~8h. Results were similar to that from a recent study in Ethiopia comparing DEET (*N*, *N*-diethyl-1,3-methylbenzamide) and MyggA (*p*-methane diol) and other laboratory products (20% neem oil and 20% chinaberry oil), where the mean CPT was 8h for DEET whilst an estimated 6h for MyggA [27]. Eight hours of repellency may suffice to protect against earlier vector biting both indoors and outdoors before residents take protection from insecticide-treated nets deployed indoors.

To date, 11 countries across the world are classified as having a high burden of malaria (2). In these countries malaria vector control is still based on the use of insecticides, either in the form of indoor spraying or by promoting the large-scale distribution of LLINs. These strategies can effectively reduce the number of malaria cases [28], however the major challenge is the resistance of malaria vectors to different classes of insecticides and the shifts in their feeding and resting behaviours, with the tendency of biting and resting outdoors. For example, a study in the Cascades region in Burkina Faso indicate that in addition to insecticide resistance, more than 50% of the malaria vector biting were taking place outdoors. Therefore, new and supplementary methods are urgently needed to complement these tools in the perspective of malaria elimination [29]. In accordance with the spirit of locally adapted-integrated vector and disease control [30], repellents can usefully complement existing control strategies and provide an additional tool in the management of insecticide resistance. In the context of widespread resistant vectors to insecticide and the tendency of mosquitoes to bite outside houses, there is a need to add MAA ointment to vector control tools in sub-Saharan, malaria-burdened countries.

The originality of MAA comes from its formulation based on local butter extensively used in rural areas of West African, and which contributes to womens economic income. Promotion of use of local endogen strategies can sustain malaria control, and also improve the economic situation for African women. Additionally, it has been shown that shea butter is a source of anti-inflammatory and anti-tumour promoting compounds [31]. Another interesting compound in shea butter is cinnamic acid which is known for its antibacterial, antifungal and antiviral properties [32]. Shea butter both moisturizes and heals the skin. Clinical studies have shown it to be safe for skin [33]. MAA ointment will not only protect from mosquito-borne diseases but also against other micro-organisms.

Besides the level of protection offered by repellents, daily compliance and appropriate use seem to be major obstacles to achieving the potential impact on malaria [34]. An efficacy study carried out in Tanzania has shown that volunteers preferred MAA ointment to a more classical 20% DEET solution [35]. Sales by Maa Africa SAS of 50,000 units in local stores in Burkina Faso between August and November 2021 further illustrate its desirability. However, more data are needed to understand who is likely to use the product and whether its usage is appropriate in terms of frequency and application in order to have an impact on mosquito-borne diseases, such as malaria.

## Conclusions

MAA, a novel ointment formulated with shea butter widely used in West Africa to moisturize the skin of children, has shown high repellency against laboratory-reared and wild malaria vectors. In the context of widespread vector resistance to insecticide and growing tendency of mosquitoes to bite outside houses, there is a need to add MAA ointment to the vector control tools used in sub-Saharan countries with high malaria burden.

## Data Availability

Not applicable.
